# Long-standing inferior vena cava filter with multidirectional strut penetration: A case report

**DOI:** 10.1016/j.radcr.2026.04.060

**Published:** 2026-05-27

**Authors:** Nawaf Salah Ayad Mohamed, Osama Osman Ahmed Farah, Mohammed Ibrahim Elhamaky, Abdulaziz Mubarak Almasan, Abdulrahman BinSwilim, Talal Mislat Alotaibi

**Affiliations:** a College of Medicine, AlMareefa University, Riyadh, Saudi Arabia; b Al-Iman General Hospital, Riyadh, Saudi Arabia; c King Salman Hospital, Riyadh, Saudi Arabia

**Keywords:** Inferior vena cava filter, Strut penetration, Fractured strut, Filter retrieval, Case report

## Abstract

Inferior vena cava (IVC) filter penetration is an increasingly recognized complication, particularly after prolonged dwell time or failed retrieval. Although often asymptomatic, multidirectional penetration into adjacent structures may pose a significant risk. We report a 61-year-old man presenting with non-traumatic radicular back pain whose CT imaging demonstrated a retrievable IVC filter placed nine years earlier with extensive strut penetration into the L3 vertebral body, perivertebral soft tissues, bowel loops, distal abdominal aorta, and right mesocolon, along with one fractured strut. Prior retrieval attempts had failed due to filter tilt and hook embedding, without evidence of hemorrhage or retroperitoneal leakage. The patient was referred for complex endovascular retrieval. This case underscores the importance of structured surveillance, timely retrieval, and specialized multidisciplinary management to prevent severe delayed complications.

## Background

Venous thromboembolism, including pulmonary embolism and deep vein thrombosis, is a significant complication after orthopedic surgery, with mortality and morbidity rates from 5% to 30%. Anticoagulation therapy is the primary prevention method; however, some patients cannot receive anticoagulants because of increased bleeding risk or recurrent thromboembolic events. In these cases, prophylactic retrievable inferior vena cava (IVC) filters may be used and are intended for removal once the risk of pulmonary embolism decreases [1]. Filter use has been increasingly associated with complications, such as strut fractures and perforations. Although most penetrations are asymptomatic, severe outcomes like hemorrhage, arrhythmia, or death have been reported [2]. Retrieval rates for these filters remain low, often due to technical difficulties, prolonged dwell times, and poor follow-up [3]. We present a case of a long-term IVC filter with significant multidirectional penetration, found incidentally, highlighting the dangers of prolonged indwelling filters and the need for careful monitoring.

## Case Report

A 61-year-old obese male with uncontrolled hypertension presented with one week of worsening lower back pain radiating to the right hip, along with numbness over the right great toe. He denied lower limb weakness, bowel or bladder incontinence, recent trauma, or heavy lifting. His surgical history included intramedullary nail fixation of the right femur 9 years ago and right humerus and knee fixation eight years ago after a road traffic accident.

On examination, the patient was alert (Glasgow Coma Scale 15/15) with blood pressure 178/133 mmHg. Neurological assessment showed decreased sensation in the right L5 dermatome, preserved S1 sensation, and full motor strength (5/5) in both lower limbs. The straight-leg raise test was positive on the right side. A healed scar was noted over the right lateral thigh and anterior knee. Laboratory tests showed mildly elevated creatinine, hyponatremia, and increased liver enzymes; all other results were unremarkable.

**Fig. 1:**
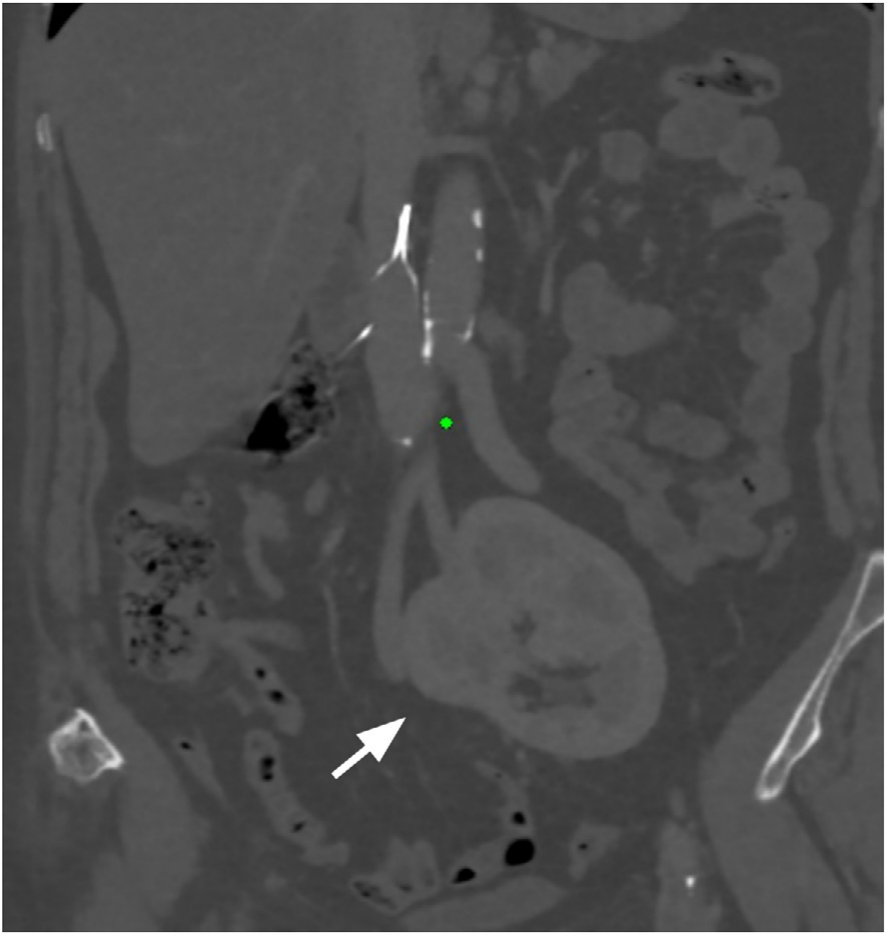
Coronal non-contrast CT kidney–ureter–bladder image demonstrating an incidental fused ectopic pelvic kidney without obstruction.

Non-contrast CT of the lumbar spine showed preserved alignment and mild-to-moderate L4–L5 posterior disc bulging, causing relative central canal stenosis, with minimal bulging at L3–L4. CT Kidney Ureter and Bladder (KUB) scan incidentally revealed an ectopic fused pelvic kidney in the true pelvis without obstruction or calculi (Fig. 1).

**Fig. 2:**
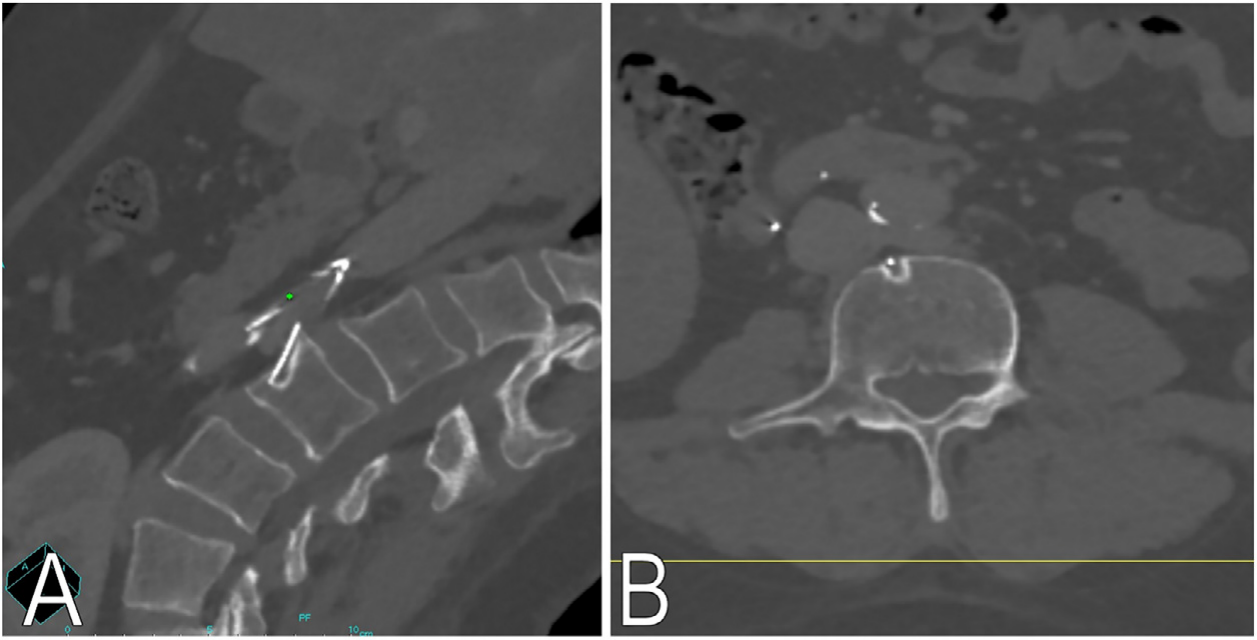
Multi-projection reconstruction images oriented (A) and axial non-contrast CT images (B) showing a filter strut penetrating the anterior L3 vertebral body with chronic cortical scalloping.

**Fig. 3:**
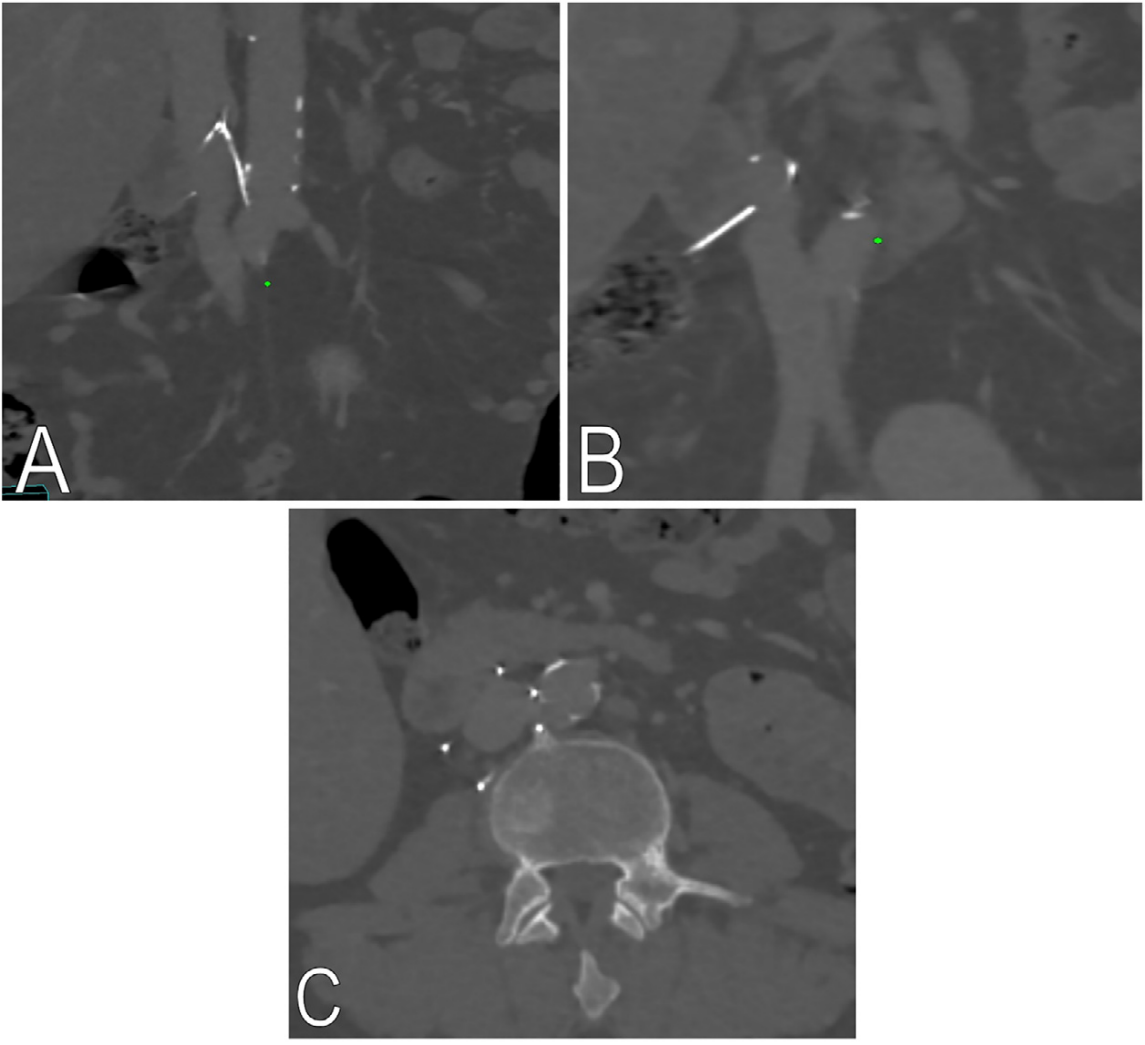
Multi-projection reconstruction images oriented (A and B) and axial CT reconstructions (C) demonstrating multi-directional IVC filter strut penetration. Two struts are adjacent to bowel loops, one strut is embedded in the distal abdominal aorta at the aortoiliac junction, and one fractured strut is projecting outside the right mesocolon.

CT of the abdomen and pelvis also revealed a retrievable IVC filter placed nine years earlier by a non-interventional radiologist at another private hospital. The patient had been told that the filter was likely harmless at that time, so no further attempts at removal were made. Multiple retrieval attempts at that hospital had failed, including one 4 years earlier due to a tilted, embedded filter hook, after which the device was deemed irretrievable and left in place. CT imaging showed multidirectional strut penetration: a strut entered the anterior L3 vertebral body, causing cortical scalloping; another was adjacent to the perivertebral region; two were near bowel loops; one was in the distal abdominal aorta at the aorto-iliac junction; and a fractured strut projected outside the right mesocolon (Fig. 2, Fig. 3). No active hemorrhage, aneurysm, or retroperitoneal leakage was identified. The patient was referred to a tertiary interventional radiology center with full multidisciplinary backup support, including vascular surgery, for assessment and planning of a complex laser-assisted filter retrieval procedure, in accordance with institutional policy for high-risk elective procedures. Given the significant procedural risks, the clinical team considered watchful waiting as a reasonable alternative until the patient’s decision. At the time of this report, the patient is still deliberating whether to proceed.

## Discussion

IVC filters have a basket-like structure that captures venous emboli from the lower limbs and prevents pulmonary embolism. Minor strut penetration into the caval wall is expected for anchorage, but extension beyond the IVC lumen by more than 3 mm is considered pathologic and may lead to complications. The main indications for IVC filter placement are when anticoagulation is contraindicated, ineffective, or associated with recurrent thromboembolic events [4,5]. Retrievable IVC filters are commonly used and generally have a favorable safety profile, but major complications such as filter fracture, migration, and penetration of adjacent structures can occur [6]. Despite widespread use, retrieval rates remain low. Prolonged dwell time is also strongly associated with increased risk of complications, including fracture, migration, penetration, and thrombosis. Retrievable filters are generally recommended for removal once the risk of pulmonary embolism subsides, given increasing reports of filter migration and strut penetration. Many penetrations remain clinically silent and are found incidentally on imaging for unrelated reasons, as in our case [7]. The literature describes a broad spectrum of penetration severity. Asymptomatic cases with duodenal or organ involvement have been reported, sometimes discovered months to years after placement [8]. Severe symptomatic cases involving cardiac, diaphragmatic, or aortic penetration are less common but can be life-threatening [9,10]. Our patient had extensive multi-directional strut penetration, including vertebral body involvement, abutment of the abdominal aorta, and a fractured strut projecting into the mesocolon, yet remained asymptomatic regarding the filter. The radicular back pain prompting evaluation was unrelated. The severity of penetration likely reflects a combination of prolonged 9-year indwelling time, multiple failed retrieval attempts, and progressive embedding of the filter hook. Several clinical considerations arise from this case: long-term IVC filters, even those initially considered safe and retrievable, carry a risk of delayed multi-structural complications; such complications may remain silent or present with atypical symptoms; CT imaging is critical for detection and characterization; and multidisciplinary planning, especially with interventional radiology, is essential for complex retrievals, although removal may not always be possible when struts involve vital structures. Limitations include the single-patient design, lack of longitudinal imaging, and incomplete procedural details regarding initial placement and prior failed retrievals. Only CT KUB and abdominal-pelvic imaging were performed, which may have underestimated organ involvement. Long-term follow-up remains unavailable, as the patient has been referred to a higher-level center and has not yet decided whether to proceed with retrieval.

## Conclusion

This case underscores the importance of long-term surveillance of indwelling IVC filters. Strut penetration can occur silently, involve multiple adjacent structures, and present with atypical symptoms. Optimal filter deployment, close follow-up, early recognition of complications, and involvement of specialized endovascular teams are essential to minimize serious adverse events and ensure safe retrieval when indicated.

## Data availability

All data generated or analyzed during this study are included in this published article.

## Author contributions

**Conceptualization:** A.B., T.M.A. **Data Curation:** N.S.A.M., O.O.A.F., M.I.E., A.M.A. **Writing – Original Draft Preparation:** N.S.A.M., T.M.A. **Writing – Review & Editing:** N.S.A.M., O.O.A.F., M.I.E., A.M.A., A.B., T.M.A. **Supervision:** O.O.A.F., M.I.E., A.M.A., A.B., T.M.A.

## Patient consent

Written informed consent was obtained from the patient for publication of this case report and accompanying images.
